# The effect of oral supplementation with a combination of beta-hydroxy-beta-methylbutyrate, arginine and glutamine on wound healing: a retrospective analysis of diabetic haemodialysis patients

**DOI:** 10.1186/1471-2369-14-8

**Published:** 2013-01-12

**Authors:** Savas Sipahi, Ozkan Gungor, Mehmet Gunduz, Mehmet Cilci, Mustafa Cahit Demirci, Ali Tamer

**Affiliations:** 1 Clinic of Nephrology, Sakarya University Training and Research Hospital, Adnan Menderes Cad. Saglik Sok. No: 193, Adapazari, Sakarya, Turkey; 2 Department of Nephrology, Ege University Faculty of Medicine, Izmir, Turkey; 3 Clinic of Internal Diseases, Yozgat State Hospital, Yozgat, Turkey; 4 Private Nefromed Hemodialysis Center, Sakarya, Turkey; 5 Clinic of Internal Diseases, Sakarya University Training and Research Hospital, Sakarya, Turkey

**Keywords:** Diabetic foot, Haemodialysis, Oral nutrition support, Arginine, Glutamine, Hydroxy methylbutyrate

## Abstract

**Background:**

Diabetes is an important reason for end-stage renal failure and diabetic foot wounds worsen the life qualities of these patients. Protein and amino acid support accelerates the wound healing. The purpose of this retrospective study is to examine the effect of beta-hydroxy-beta-methylbutyrate, arginine and glutamine (Abound®) supplementation on the wound healing.

**Methods:**

A total of 11 diabetic dialysis patients were included in this retrospective study aiming to evaluate the effect of the diet support with beta-hydroxy-beta-methylbutyrate, arginine and glutamine on wound healing in diabetic dialysis patients. Pre-treatment and post-treatment wound depth and wound appearance were scored in accordance with the “Bates-Jensen” wound assessment tool. The results of 4-week treatment with beta-hydroxy-beta-methylbutyrate, arginine and glutamine (Abound®) support were evaluated in terms of wound healing.

**Results:**

The mean age of patients was 66 (SD: 10, range: 51-81) and 9 (81.8%) of them were males. After the 4-week treatment, in accordance with the Bates-Jensen scoring, healing was observed on the wound depth score of 7(63.6%) patients and on wound appearance score of 8(72.7%) patients out of 11. While the wound depth score of 4(36.4%) cases and wound appearance score of 3(27.3%) cases remained the same, no deterioration was observed on any cases throughout the follow-up period.

**Conclusion:**

In conclusion, our findings revealed that Abound treatment makes a positive contribution to the wound healing in diabetic dialysis patients.

## Background

Today, diabetes and chronic glomerulonephritis are still among the most frequent reasons of the end-stage renal failure. More than 20% of patients receiving the dialysis treatment are diabetic [1]. Microvascular and neurological complications including retinopathy-related visual impairment and peripheral neuropathy that develop among the diabetic patients adversely affect the quality of life, especially in the dialysis patients.

Foot ulcer is one of the most frequently encountered complications in diabetic dialysis patients with peripheral neuropathy and ischemia. Diabetic foot ulcers are known to become frequently infected which leads an increase in the risk of amputation unless treated at earlier stages [2]. Both the circulation failure and impaired immune response in diabetic dialysis patients have negative effects on the wound healing. Characterized by a dynamic and complex process that involves phases of inflammation, proliferation, and remodeling that lead to the restoration of cellular structures and tissue layers [3], wound healing depends on adequate nutrient flow with substantial role of early aggressive nutrient and micro-nutritional feeding in the control and prevention of this process [4]. In this regard, essential amino acids play a significant role in wound healing in relation to their role in the production of inflammation and fibroblast products. Arginine is one of the most important amino acids that accelerate the wound healing [5] while glutamine is defined as “situational essential amino acid” which is used by fibroblasts as the primary energy resource for proliferation [6]. Hydroxy methylbutyrate (HMB) is the leucine metabolite, which is an essential amino acid [7] shown to have positive effects on the wound healing [8]. Although beta-hydroxy-beta-methylbutyrate, arginine and glutamine (Abound®) support is known to accelerate the wound healing process [9], there is limited information regarding the case in diabetic dialysis patients.

The purpose of this retrospective study is to evaluate the effect of the diet support with beta-hydroxy-beta-methylbutyrate, arginine and glutamine on wound healing in diabetic dialysis patients.

## Methods

### Study population

A total of 11 diabetic dialysis patients were included in this retrospective study aiming to evaluate the effect of the diet support with beta-hydroxy-beta-methylbutyrate, arginine and glutamine on wound healing in diabetic dialysis patients. Cases treated with haemodialysis due to chronic renal failure (CRF) and receiving oral supplementation with Abound® due to diabetic foot wound were included in the present study, while patients who were treated with antibiotics for long periods, received steroid treatment or hyperbaric oxygen therapy were excluded.

As a part of routine practice in the study centre, written informed consent was obtained from all patients considering use of their photos for any possible scientific publication in the future. The study which was conducted in accordance with the ethical principles stated in the “Declaration of Helsinki”. Additionally, while the present study was exempt from the requirement of ethical approval in relation to its retrospective design, the permission was obtained from our institutional ethics committee (Clinical Research Ethics Committee of Sakarya University) for the use of patient data for publication purposes.

### Assessments

Data were extracted from the institutional database “Nefromed Dialysis Center” database (Sakarya, Turkey) including laboratory analyses performed by “System Medical Labs” (Istanbul / Turkey). Data on demographics and basic clinical and laboratory data of selected patients who commenced their treatment between May-August 2011were recorded before the treatment. The pre-treatment and post-treatment wound depth and wound appearance of patients were scored in accordance with “Bates-Jensen” wound assessment tool shown in Table 1[10]. Photos of all of the lesions were taken both before and after the treatment. The results of 4-week treatment with beta-hydroxy-beta-methylbutyrate, arginine and glutamine (Abound®) supplementation were evaluated in terms of wound healing. Pre and post treatment values were compared with either paired t test or Wilcoxon test regarding distribution pattern.

**Table 1:** “Bates-Jensen” wound assessment tool

**Wound depth score**		**Wound appearance score**	
Necrotic	1	Necrotic	1
Deep	2	Scabbed	2
Moderate	3	Granulated	3
Easy	4	Epithelial	4
Minimal	5	Closed	5

### Treatment

The patients received the oral supplementation with a combination of beta-hydroxy-beta-methylbutyrate, arginine and glutamine (Abound®) twice the same mixture per day for 4 weeks as follows; the bags containing 1.3 g HMB, 7.4 g arginine, and 7.4 g glutamine with water of 120 cc day and night.

## Results

### Demographics, basic clinical and laboratory characteristics

Patient demographics revealed that the mean age of patients included in the study (n=11) was 66 years (SD: 10, range: 51-81) while males composed 81.8% (n=9) of the study population (Table 2).

**Table 2:** Patient demographics and basic clinical and laboratory findings

	**Mean(SD)**	**Range**	
Age (years)	66 (10)	51-81	
Gender (male,%)	81		
**Clinical/laboratory findings**	**Pre-treatment**	**Post-treatment**	**p value**
	**Mean(SD)**		
SBP (mmHg)	128(12)	130.0(9.0)	0.60
DBP (mmHg)	86 (10)	88.0(10.0)	0.62
HbA1_c_ (%)	6.6(0.9)	6.5(1.4)	0.85
CRP (mg/dl)	8.9(5.8)	9.7(7.8)	0.81
Albumin (gr/dl)	4.0(0.3)	4.1(0.0)	0.89
Hemoglobin (gr/dl)	11.7(2.0)	11.5(2.0)	0.79
WBC (10^3^/μL)	6.9(1.7)	6.2(1.3)	0.3
Urea (mg/dl)	159.0(49.0)	146.0(47.0)	0.56
Creatinine (mg/dl)	6.6(1.7)	6.5(2.1)	0.89
Total cholesterol (mg/dl)	159.0(37.0)	166.0(53.0)	0.75
Triglyceride (mg/dl)	192.0(162.0)	249.0(214.0)	0.54
ALT (U/L)	12.4(2.0)	14.9(2.6)	***0.04***
Na (mmol/L)	142.0(2.0)	139.0(2.0)	***0.01***
K (mmol/L)	5.5(0.3)	5.2(0.8)	0.33

SBP: systolic blood pressure; SD: standard deviation; DBP: diastolic blood pressure; CRP: C-reactive protein (CRP); ALT: alanine aminotransferase; Na: sodium; K: potassium.

The mean (SD) hemodialysis (HD) period of patients was 65(8) months. A total of 10 patients for the brachial arterio-venous fistula and 1 patient for the permanent jugular haemodialysis catheter took the low-flux haemodialysis treatment. Nine patients had been on long+short acting insulin treatment.

According to basic clinical and laboratory data of patients as illustrated in Table 2, there was no significant influence of treatment on clinical and laboratory findings except for increase in alanine aminotransferase (ALT, U/L; 12.4(2.0) vs. 14.9(2.6), p=0.04) and decrease in sodium (Na, mmol/L, 142.0(2.0) vs. 139.0(2.0), p=0.01) levels. Accordingly, the mean level for HbA1c was 6.6 (SD: 0.4, range: 6-8.2) and 6.5(SD: 1.4, range: 5.4-9.5) before and after the treatment, respectively. The mean (SD) C-reactive protein (CRP) levels of patients were 8.9(5.8) mg/dl and 9.7(7.8) mg/dl before and after the treatment, respectively (p=0.81).

### Treatment outcome

**Figure 1:**
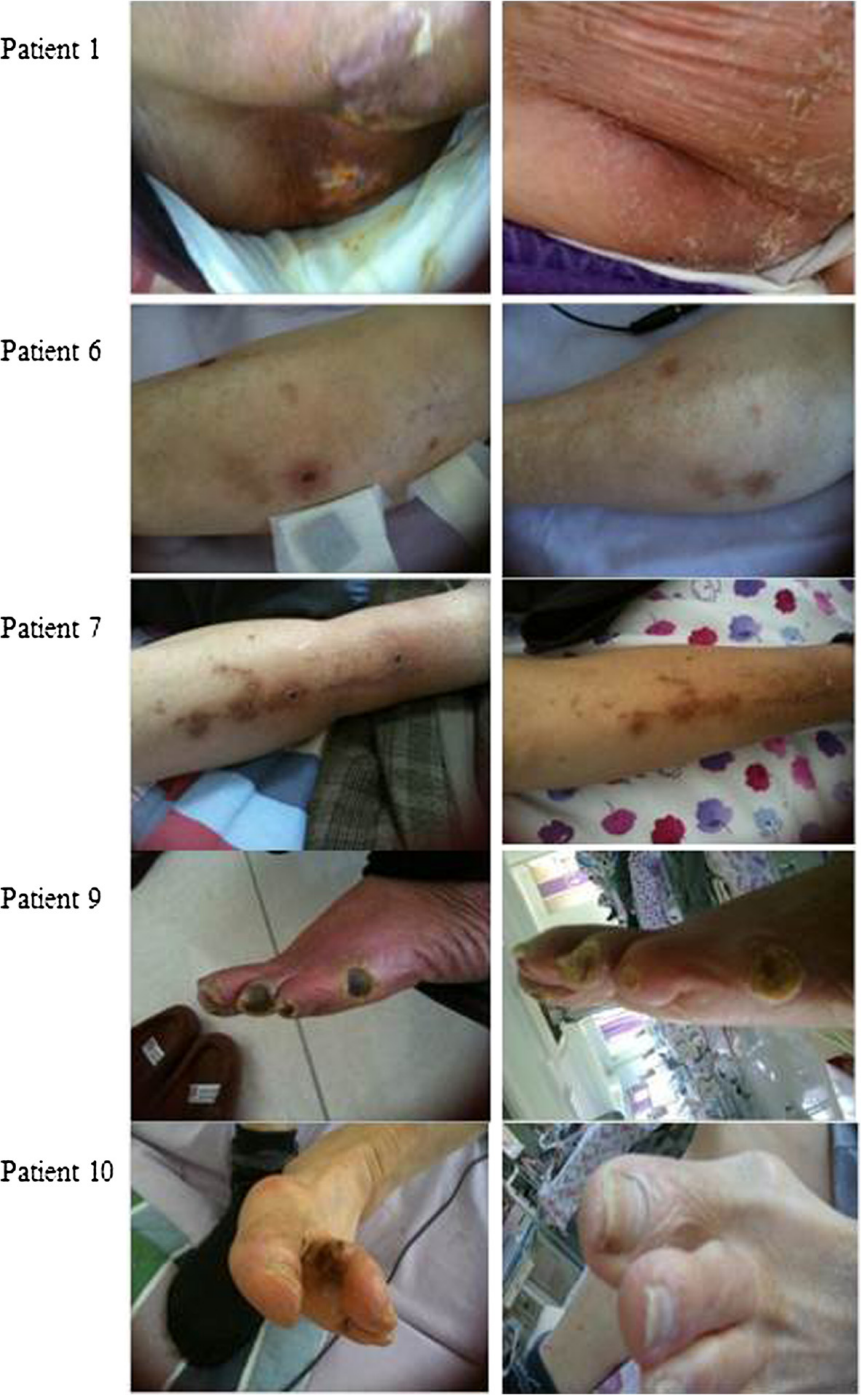
Pre-treatment and post-treatment wound appearances of the selected patients.

After the 4-week treatment, in accordance with the Betas-Jensen scoring, healing was observed on the wound depth score of 7(63.6%) patients and on wound appearance score of 8(72.7%) patients out of 11. While the wound depth score of 4(36.4%) cases and wound appearance score of 3(27.3%) cases remained the same, no deterioration was observed on any cases throughout the follow-up period (Table 3, Figure 1). Wound appearance (p=0.001) and depth (p=0.006) scores improved significantly with treatment (Table 4). The drug was tolerated well by the patients and no side effect was observed regarding the drug.

**Table 3:** Pre-treatment and post-treatment wound appearance and depth scorings of patients

	**Wound appearance score**		**Wound depth score**	
	**Pre-treatment**	**Post-treatment**	**Pre-treatment**	**Post-treatment**
Patient 1	1	5	2	4
Patient 2	5	5	5	5
Patient 3	3	4	4	5
Patient 4	1	3	1	3
Patient 5	2	4	2	3
Patient 6	4	5	4	5
Patient 7	2	5	3	5
Patient 8	2	4	3	5
Patient 9	3	5	3	5
Patient 10	2	2	3	4
Patient 11	3	3	3	3

**Table 4:** The effect of treatment on wound appearance and depth score

	**Pre-treatment**	**Post-treatment**	**p value**
	**Median(IQR)**		
Wound appearance score	2.0(1.0)	4.0(2.0)	***0.011***
Wound depth score	3.0(2.0)	5.0(2.0)	***0.006***

IQR: inter-quartile range.

## Discussion

Diabetes is one of the most frequent reasons of end-stage renal failure and a well-established cause of the development of foot lesions [3]. Almost 25% of patients with diabetes have been stated to develop foot ulceration as a complication during their lifetime [11]. Furthermore, independent of diabetes, renal failure has been reported to markedly increase the risk of diabetic foot ulceration and amputation [3,12]. Hence the coexistence of these two conditions, there is a double threat for development of foot lesions which impair the patient’s quality of life, increase health care costs, and, for patients who require surgical interventions, there is a higher mortality compared with patients without renal disease [3].

Given that any significant wound leads to a hyper-metabolic and catabolic state, wound healing is a complex chain of biochemical events during which nutritional needs are significantly increased [4]. It is a well-known fact that protein and amino acid support accelerates the wound healing. In this retrospective study, it was determined that the nutrition support combination consisting of beta-hydroxy-beta-methylbutyrate, arginine and glutamine would be useful for the treatment of foot ulcers, which are encountered as an important problem in diabetic dialysis patients.

Due to its rapid metabolism, the wound needs high amounts of nutritional elements while protein-calorie malnutrition extends the phase of inflammation with negative effects on the fibroplasia, proteoglycans and collagen synthesis. Essential amino acids play a significant role in wound healing by increasing the production of inflammation and fibroblast products [13]. Beta-hydroxy-beta-methylbutyrate is a leucine metabolite. It decreases the proteolysis, increases the protein synthesis, decreases the apoptosis and increases the cell proliferation. HMB is generally used safely as support in patients with malnutrition, cancer, chronic disease, sepsis and HIV [14]. Being a product of the ornithine metabolism, arginine is required for the cell proliferation, regeneration and collagen synthesis [5]. Glutamine is not essential for the body; however, it is the most intense amino acid in the body that regulates the cell functions, coordinates the immune parameters, displays an anti-oxidant effect and regulates the adjuvant T-cell functions [6]. In a study by Arana et al. evaluation of the effect of arginine treatment on healing of foot ulcer in 11 diabetic patients revealed that the recovery was complete for 8 patients and considerable for 3 patients [15]. Costa et al. indicated that glutamine support significantly improved the laceration strength of wound and increased the percentage of mature collagen area [16]. Likewise, HMB is known to have positive effects on wound healing.

Abound is a special formula that is consisted of beta-hydroxy-beta-methylbutyrate, arginine and glutamine that supports the wound healing and enables the structuring of lean body mass. This combination is potentially used among patients with cancer, sarcopenia, trauma, chronic diseases, chronic obstructive lung disease and HIV [17,18]. However, the studies previously conducted have concluded that this product also accelerates the wound healing. In their study that was performed on 18 healthy adults older than 70 years of age, Williams et al. indicated that the Abound support increased the collagen synthesis and accelerated the wound healing [19]. The period of ulcer healing was also shown to be significantly shortened with by Tatti et al. in retrospective evaluation of 12 patients with diabetic foot who were receiving the Abound treatment [20]. Likewise, our findings also indicated that the healing was observed on wound depth score of 7 patients and on wound appearance score of 8 patients out of 11. While the wound depth score of 4 cases and wound appearance score of 3 cases remained the same, no deterioration was observed on any cases throughout the follow-up. In line with no negative side effect associated with the Abound treatment in the literature, none of our patients experienced a negative effect throughout the treatment.

The major limitation of the present study is its retrospective design as an uncontrolled series of diabetic haemodialysis patients besides the small sample size. Nevertheless, given that malnutrition is a common and well-known deleterious outcome in dialysis patients which delays wound healing, our findings indicating that beta-hydroxy-beta methylbutyrate, arginine and glutamine may be very beneficial in these patients by not only improving wound healing seem notable.

## Conclusion

In conclusion, our findings indicate the benefit of beta-hydroxy-beta-methylbutyrate, arginine and glutamine support in wound healing including diabetic foot among diabetic dialysis patients. Nevertheless, given the small sample size as well as retrospective design of the present study, future larger-scale prospective studies would be warranted to conclude the efficacy of beta-hydroxy-beta-methylbutyrate, arginine and glutamine supplementation in wound healing among diabetic dialysis patients.

## Abbreviations

CRF: Chronic renal failure; CRP: C-reactive protein; HD: Hemodialysis; HMB: Hydroxy methylbutyrate.

## Competing interests

The authors declare that they have no competing interests.

## Authors’ contributions

SS carried out study concept and design, and study supervision, OG carried out drafting of the manuscript, MG carried out analysis and interpretation of data, MC carried out acquisition of data, MCD carried out acquisition of data, AT carried out critical revision of the manuscript for important intellectual content. All authors read and approved the final manuscript.

## Pre-publication history

The pre-publication history for this paper can be accessed here:

http://www.biomedcentral.com/1471-2369/14/8/prepub
